# Comparing habit-behaviour relationships for organised versus leisure time physical activity

**DOI:** 10.24072/pcjournal.311

**Published:** 2023-08-31

**Authors:** Katerina Newman, Cyril Forestier, Boris Cheval, Zachary Zenko, Margaux de Chanaleilles, Benjamin Gardner, Amanda L. Rebar

**Affiliations:** 1 Motivation of Health Behaviours Lab, Appleton Institute, Central Queensland University – Rockhampton, Australia; 2 Laboratoire Motricité, Interactions, Performance, MIP - EA4334, Le Mans Université – Le Mans, France; 3 Department of Sport Sciences and Physical Education, Ecole normale supérieure Rennes – Bruz, France; 4 Laboratory VIPS2, University of Rennes – Rennes, France; 5 California State University – Bakersfield, United States of America; 6 Laboratoire SENS, Univ. Grenoble Alpes – Grenoble, France; 7 Habit Application and Theory group, Health Psychology Research Group, School of Psychology, University of Surrey – London, United Kingdom

## Abstract

Evidence shows that people with strong physical activity habits tend to engage in more physical activity than those with weaker habits, but little is known about how habit influences specific types of physical activity. This study aimed to test whether mean level of habit strength and magnitude of the habit strength – behaviour association differed as a function of physical activity modality. Participants (N = 120; M age = 25 years, 75% female) who reported engaging in organised sport separately reported their habit strength for organised sport and leisure time physical activity as well as the time they spent engaging in these physical activity behaviours. Means comparisons and multilevel modelling revealed that people had significantly stronger habit for organised sport than for leisure time physical activity. Crucially, no significant difference was found in the magnitude of the sport-habit and leisure-habit link. Post-hoc analyses revealed that habit was stronger for team sport compared to individual sport, but that there was no significant difference in sport-habit association between team and individual sports. Research should therefore focus on identifying the characteristics of team sports-based activity that are particularly conducive to habit formation as a precursor to developing interventions to promote performance of leisure time activity in a way that would attain such characteristics.

## Introduction

Tackling major non-communicable diseases is of global importance for reducing mortality and providing better health outcomes for individuals of all ages (Guthold et al., 2018). Regular physical activity is effective in the prevention of chronic diseases such as diabetes, cancer, hypertension, cardiovascular disease, obesity, depression and osteoporosis (Penedo & Dahn, 2005; Warburton et al., 2006). The contribution of physical inactivity to non-communicable diseases is the fourth leading cause of death worldwide, contributing to 5.3 million preventable deaths annually- or one death every 6 seconds worldwide (Bull et al., 2020; World Health Organisation, 2020). Globally, low physical activity engagement rates have remained mostly stable for the past two decades (World Health Organisation, 2020), despite ongoing public health efforts to increase physical activity. Clearly, a more refined understanding of the influences that motvate regular engagement in physical activity is needed.

Because many of the health benefits of physical activity rely on maintained behavioural engagement, habit may be an important motivational construct to consider. Although habit has traditionally been viewed as frequently repeated behaviour, amongst psychology, habit is more commonly defined not as a behaviour, but instead as a cognitive or psychological precursor to behaviour (Hagger & Rebar, 2020; Rhodes et al., 2010; Rebar et al., 2018; Gardner, 2015). Habit is defined as a process by which a stimulus generates an impulse to act as a result of a learned stimulus-response association. After regularly engaging in the same physical activity within the same context, a person can develop strong physical activity habits. For example, if every Sunday afternoon, a person participates in a neighbourhood basketball game, the decision of whether to participate in the game each week becomes less of a deliberation and more of a habitual, automatic response. Similarly, if a person routinely walks their dog each morning after breakfast, this can become habitual response over time, resulting in more consistent engagement in that leisure time physical activity behaviour over the long-term.

Theoretically, the premise is that physical activity can become habitual, in which the habitual behaviour is rooted in associative memory and practised automatically, independent of goals and intentions (Hagger, 2018). Habit-generated influences on physical activity behaviour may compete or combine with other motivational sources, including conscious decision-making, to influence behaviour (Gardner, 2015). There is a large body of evidence establishing that people with strong physical activity habits tend to engage in more physical activity behaviour than people with weaker physical activity habit (Hagger, 2018; Rebar, 2017).

Physical activity habit research has generally focussed on the broad behaviour construct of physical activity and asking people about the strength of their habits “for physical activity” globally (e.g., Gardner et al., 2011; Rebar et al., 2016). ‘Physical activity’ as a blanket term fails to account for the vast differences in what these activities can involve, and the levels of energy and intention required to exert them. That is, many different behaviours could be considered as ‘physical activity’– anything from training with teammates for a competition to walking to the nearby bus stop to gardening on the weekend. There are significant between-person differences in which types of physical activities people prefer and most commonly engage in (e.g., Jago et al., 2005; Li et al., 2017; Slater & Tiggemann, 2011). It may also be that people have different habits for different types of physical activities.

Whereas some people tend to engage in non-organised related leisure time physical activity such as running, hiking, or jogging; others prefer organised sport including basketball, soccer, or tennis. People’s planning and preparing efforts for leisure time physical activity versus organised sport are likely quite different, so the habitual nature of these modalities in particular are worth comparing. Leisure time physical activity is non-sport related exercise or activity that takes place in a person’s free time and is considered non-essential physical activity performed at the discretion of the individual solely for recreation, exercise, or leisure (Booth, 2000). Organised sport, on the other hand, tends to be a scheduled event that people have committed to long-term (e.g., seasonal) commitments to a team or club. To develop into strong habit, behaviours need to be repeated with regularity within a specific context (e.g., in same part of routine, same time of day, around same people, in the same location) so that the cue-behaviour relationship can develop and strengthen (Lally et al., 2010; Verplanken, & Melkevik, 2008). Occurring, by definition, in our free-time, leisure time physical activity is less likely to be highly structured and can change from day-to-day or week-to-week depending on various factors: families, work commitments and the schedules of the other people. Based on the theoretical premise that cue-behaviour consistency is essential for the development of habit, it could be expected that leisure time physical activity may be less susceptible to habit formation than organised physical activity. Although there may be anticipated differences in the overall strength of leisure time vs organised physical activity habit strength, there is no theoretical premise to suggest that habit has a different level of influence on different types of physical activity behaviour. Although in line with habit theory, these notions have never before been tested.

### The Present Study

To extend the body of evidence investigating how physical activity behaviour of any kind is associated with the strength of habit, this multinational study aimed to provide insight into whether habit strength and the habit strength – behaviour association differed as a function of type of physical activity modality. We hypothesized that habit would be stronger overall for sport than for leisure time physical activity (H1), and that the strength of the association between habit strength and behaviour would not significantly differ between organised sport and leisure time physical activity (H2).

## Methods

### Procedures

Data for this cross-sectional survey study were collected from the UK, USA, Australia, and Switzerland in 2020. Participants were recruited through email lists, social media posts, and student participant pools. The survey was open to all participants who had access to a PC or laptop and were older than 18 years, with the exception that it was open to those 16 years or older in the United Kingdom. Participants in the United Kingdom, United States, and Australia who completed the study were offered a gift voucher worth £7, US $10, and AU$10, respectively. Participants in Switzerland were instead offered course credit for participation. Participants were provided with a link to the survey to provide informed consent and participate in the study. The study was hosted on Inquisit Millisecond 6.2® in English (United Kingdom, United States, and Australia) and French (Switzerland).

All study procedures were approved by the institutions’ ethical boards prior to the study UK (MRSU-20/21-21217), US (Protocol 21-178), Australia (Central Queensland University’s Human Research Ethics Committee, Project #22643) and Switzerland (CCER-2019-00065).

### Participants

An a priori power analysis was conducted to determine what sample size would be needed to find small-medium sized mean differences in habit strength between organised sport and leisure time physical activity (Champley, 2020). The analyses revealed that for a paired sample t-test, a study with at least 84 participants would be sufficiently powered (1 - β = 95) for a mean difference = .40. at a significance level of .05. The anticipated mean difference was based on the range of values of physical activity habit strength found in past research (Gardner et al., 2011; Rebar et al., 2016).

Data from 308 participants who provided consent for the final study were used. To be included in the study, participants were to be aged 18 years or older (16 years or older for the UK), and self-reported engaging in organised sport. However, given the aim of the analyses for this study was to compare habit strength of sport and leisure-time physical activity within-person, participants were excluded if they did not report practicing sport in a club or competition.

### Measures

#### Demographic Factors

Participants were asked to self-report their age in years and their gender was asked through the sentence “what best describes your gender?”. Participants could select from options “male”, “female”, “I use another term”, “prefer not to say”.

#### Leisure Time Physical Activity and Organised Sport Habit Strength

Leisure time physical activity and organised sport habit strength were measured by the Self-Report Behavioural Automaticity Index (Gardner et al., 2012), a four-item subscale of the Self-Report Habit Index (Verplanken & Orbell, 2003) that assesses the extent to which the initiation of a behaviour is automatic. Participants were asked to indicate the degree of automaticity involved in deciding to engage in leisure time physical activity/sport, such as “the decision to [do moderate to vigorous physical activities in my free time / play sport] is something I do without thinking”. Participants responded to each item on a 7-point Likert-type scale (1 never/strongly disagree – 7 always/strongly agree). Responses to the four items within each scale were averaged to create a score for leisure time physical activity habit strength and a score for organised sport habit strength, with higher scores indicative of stronger habit (leisure time physical activity habit α = .83, ω = .88; organised sport habit α = .83, ω = .92).

#### Leisure Time Physical Activity and Organised Sport Behaviour

Leisure time physical activity behaviour and organised sport behaviour were measured using adaptations of the validated short version of the International Physical Activity Questionnaire (Craig et al, 2003). For leisure time physical activity, participants were asked to self-report the amount of time (per week in minutes) spent completing “moderate to vigorous physical activities during free time”, with moderate activities described as those which “require moderate physical effort and make you breathe somewhat harder than normal”, and vigorous activities those which “take hard physical effort and make you breath somewhat harder than normal”. Participants were asked not to include time spent being active moving from one place to another (e.g., transport), nor time spent playing sport in a club or competitively. We used the time spent in min per week in both moderate and vigorous physical activity as the outcome.

For organised sport behaviour, participants were asked if they participated in sport “competitively or in clubs”. Participants who engaged in organised sport were then asked to self-report “On average, how much time do you spend playing sports as part of your competitive or club sports practice only?”. The variable was calculated with this self-report of activity in minutes per week, with higher scores indicative of more organised sport behaviour.

### Data Management and Analyses

There was evidence of risk of undue influence of outliers from skew of physical activity behaviour variables. To mitigate this risk, we windsorised the variable to the third quartile. All data analyses were conducted in R Studio version 1.3.1093 (R Core Team, 2019; RStudio Team, 2020). Full R script and data are available at Rebar et al. (2023).

To test the first hypothesis that people would have stronger habit for organised sport behaviour than for leisure time physical activity, a paired samples t-test was conducted. To test the second hypothesis that the association between people’s organised sport behaviour and sport habit would be stronger than the association between people’s leisure time physical activity habit and leisure time physical activity behaviour, moderation analysis within a multi-level model was conducted (Bates et al., 2015). Specifically, the data were structured to have ‘modality’ (i.e., leisure time vs organised sport) as a nested variable within-person. The model was set with time spent in behaviour as the dependent variable, with habit strength, modality, and the mean-centred interaction term between modality and habit strength as predictors. After random effect structure testing to find the best fit for the data, random effects were set so that slopes and intercepts were allowed to vary between individuals. Age, gender, and study language (English vs French) were also included as covariates. Estimated marginal means were calculated to determine the habit-behaviour slopes for the separate modalities (Lenth, 2021). Prior to and throughout the model estimations, assumption testing was conducted with all assumptions met.

## Results

### Sample Characteristics

The final analysed sample included 120 participants who reported being involved with organised sport (M age=25.62, SD= 5.51). Most (75%) of the sample were aged 21 years or older. Slightly more than half of the sample (55%) identified as male. Most (75%) of the sample were aged 21 years or older, and 55% were male (n = 66; 43% female, n = 52; 2% not disclose), n =2). Compared to the full sample of N = 308, those who engaged in organised sport (and thus were eligible for inclusion in this analysis) were more likely to be male χ2 = 210,86, p < .01. There was no difference in age or leisure time physical activity habit strength between those who did and did not engage in organised sport (p’s > .05). Those who engaged in organized sport had significantly stronger leisure time physical activity habit strength than those who did not (M difference 95% CI = 0.36 to 1.02), but there was no difference in leisure time physical activity (p = .19).

Participants engaged in an average of 242 minutes of leisure time physical activity behaviour per week (M = 242.40, SD = 243.29) and 164 minutes of organised sport behaviour per week (M = 164.53, SD = 1.20; see Table 1). The most commonly reported sports were football, tennis, and basketball. There were no gender differences in leisure time physical activity or organised sport habit strength or behaviour (p’s > .05). There was no correlation between age and leisure time physical activity habit strength, leisure time physical activity behaviour, or organised sport behaviour; however, there was a significant inverse correlation of age with organised sport habit strength, indicating that younger people had significantly stronger organised sport habit than older people. Habit strength for organised sport was positively associated with organised sport behaviour. However, habit strength for leisure time physical activity was not significantly associated with leisure time physical activity behaviour. The two habit strength scores were moderately, positively associated, such that those with strong organised sport habit also tended to have strong leisure time physical activity habit.

### Habit Strength for Leisure Time Physical Activity and Organised Sport

It was hypothesised that participants would have stronger organised sports habits than leisure time physical activity habits. Consistent with H1, the paired t-test revealed that participants had stronger habits for organised sport (*M* = 4.30) than for leisure time physical activity (*M* = 3.89), [95% CI = 0.17 to 0.64], *t*(119)= 3.45, *p <* .01. It was also hypothesised that the strength of the association between habit strength and behaviour would not significantly differ between organised sport and leisure time physical activity (H2). The multilevel modelling results are depicted in Table 2, revealing that the H2 was not supported – there was no statistically significant difference in the habit-behaviour link between sport and leisure time modalities. This means that the association between habit and behaviour was not significantly different in magnitude between leisure time and organised sport physical activity, so hypothesis 2 was not supported. The post-hoc estimated marginal means analyses revealed that for leisure time physical activity, the behaviour-habit slope estimate was 26.00 [95% CI = 18.99 to 33.00], demonstrating a significant, positive association between habit and behaviour, and for organised sport physical activity, the behaviour-habit slope estimate was 18.70 [95% CI = 5.54 to 31.90], also demonstrating a significant, positive association between habit and behaviour. The slopes did not significantly differ from one another in magnitude (t-value = 0.99, *p* = .32). Notably, there were no significant age, gender, or study language effects on behaviour.

### Post-Hoc Analysis: Habit Strength for Team vs Individual Sport

In a post-hoc analysis, it was tested whether habit strength varied between team vs individual sports and whether the association between sport behaviour and habit strength for sport differed as a function of team vs individual sports. This was investigated in consideration of whether social aspects of sport would impact the habit-behaviour association. The sports written in via open response format were coded as generally a team (e.g., basketball, football) or individually-based sport (e.g., swimming, running). An independent t-test was calculated to test for mean differences in sport habit strength between team vs individual sports and a simple linear regression was estimated with sport behaviour regressed onto habit strength for sport, team vs individual sport (with individual sport as a reference), and the interaction term of mean-centred habit strength and team vs individual sport. Covariates of age, gender (with male as reference), and survey language (with English as reference) were included in the model. The mean comparison revealed that habit strength was statistically significantly stronger for team sports (*M* = 4.47) than for individual sports (*M* = 4.18; *t* = 54.87, *p* < .01). The results shown in Table 3 reveal that the association between sport habit and behaviour did not significantly differ as a function of whether it was a team or individual sport. Regardless, habit strength was associated with organised sport behaviour.

## Discussion

The present study findings showed that habit strength was stronger for organised sport than for leisure time physical activity, but that there was no significant difference in the degree to which habit strength was associated to behaviour. Hence, our study suggests that organised sport may be more susceptible to habit development than leisure time physical activity, but once formed, habits influence behaviour to the same extent, regardless the type of behaviours. Post-hoc analyses further support these claims revealing that the link between habit strength and behaviour did not differ as a function of team vs individual sport, but that people tended to have stronger habits for team vs individual sports.

Habit is understood to be a precursor for long-term behavioural maintenance (Lally et al., 2011) and has been established as an important motivational factor of physical activity (Hagger, 2019; Rebar, 2017). Habit formation occurs when behaviour is repeated in the same context consistently over time (Lally et al., 2010). Given that organised sports may be more likely to be repeated over and over with the same group of people and often in the same environment, we speculate that these aspects of organised sport make it likely to induce habit. In comparison, leisure time physical activity is more likely to occur sporadically and with less structure, which may make it less likely to become habitual. Future investigation should build on our findings by experimentally manipulating the aspects that distinguish organised sport from leisure time physical activity to determine precisely which facets are the key ingredient to habit formation.

We suspect that the structure and routine characteristic of most team-based sports programs may be valuable for habit formation. Repetition of behaviour in the presence of the same cues creates cue-behaviour associations, leading to strong habit (Gardner, 2015). Efforts to drive engagement in different kinds of physical activity should consider the contextual cues such as mood, person, place, and sequence in routine as well as the frequency in which (or how repetitively) they occur (Pimm et al., 2016). If efforts are made to make leisure time physical activity more cue-consistent, leisure time activity habits would be expected to become more habitual over time. The development of interventions designed to increase physical activity should therefore be mindful of contexts, frequency and scheduling as important frameworks which may facilitate better habit formation, thereby making long-term engagement much more likely to occur. Alternative explanations for the difference in habit strength between organised sport and leisure time physical activity should also be explored including the intrinsic motivation for the behaviour, investment, and strict scheduling. Given that habit strength was stronger for team vs individual sport, it could be speculated that social mechanisms could be at play such as social connectedness or accountability.

A significant association was found between habit and behaviour for both organised sport and leisure time physical activity, in line with research that suggests that habit is a psychological precursor to behaviour **(**Rhodes et al., 2010; Rebar et al., 2018). Notably, there was no statistically significant difference in the degree to which habit strength was associated with organised sport versus with leisure time physical activity behaviour or between individual vs team sports. Taken together with our finding that leisure time physical activity habits tend to be weaker, this suggests that leisure time physical activity habits, once formed, have the same power to generate behaviour in associated contexts as do habits for organised sports.

### Study Limitations

For the study, behaviour was self-reported, which can lead to systematic biases in overestimation of time spent in physical activity (Heesch et al., 2010; Lee et al., 2011). Habitual behaviours also require less deliberation to engage with, therefore may be less likely to be recalled accurately (Hyde et al., 2012). Future research should consider the use of combined monitor and self-reported modality measures to reduce response biases of physical activity. Additionally, we constrained our study to leisure time and organised sport physical activity, but further work is needed to determine how habit strength plays a role in other modalities of activity including active transport and occupational or household activity. The current study is also a cross-sectional design and therefore cannot test for causality or direction of effects. Researchers may consider the use of longitudinal and intervention designs to ensure these findings of between-person differences apply at the within-person level.

### Conclusion

To optimise maintenance of physical activity, improving the capacity for habit formation may aid interventions aimed at encouraging maintenance of leisure time physical activity for ongoing health. Our findings reveal that organised sports may be more conducive to habit formation than leisure time physical activity, but that the strength of influence of habit on behaviour is no different between these two physical activity modalities. Additionally, team sport habits tend to be stronger than individual sport habits, but again the link between organised sport behaviour and habit do not differ between spots. Work is needed to understand what makes team-based sport activity inherently ‘habit-friendly’, and to encourage performance of other physical activities in a way that is equally conducive to habit formation.

## Figures and Tables

**Table 1:** Means, Standard Deviations, and Bivariate Correlations of Study Variables

		*M*	*SD*	2.	3.	4.	5.
*1.*	*Age (in years)*	25.62	5.51	0.08	-0.18	-0.24*	-0.07
*2.*	*LTPA habit strength*	3.89	1.28	--	0.03	0.46*	0.04
*3.*	*LPTA behaviour (in min)*	242.40	243.29		--	-0.05	0.35*
*4.*	Organised *sport habit strength*	4.30	1.20			--	0.20*
*5.*	Organised *sport behaviour*	164.53	1.20				--

Note: LTPA indicates leisure time physical activity, * *p* < .05

**Table 2:** Results of Multilevel Linear Regression Analysis Testing Whether the Association between Habit Strength and Behaviour Differs Between Leisure Time Physical Activity and Organised Sport

	*b*	95% Confidence Interval
Intercept	111.25*	65.23 to 157.12
Habit strength	26.00*	19.08 to 32.92
Modality (with LTPA as reference)	-14.07	-76.25 to 48.98
Habit × Modality	-7.28	-21.85 to 7.05
Age	-0.95	-2.55 to 0.66
Gender (with male as reference)	9.95	-118.26 to 138.17
Survey language (with English as reference)	1.72	-22.85 to 7.05

Note: LTPA indicates leisure time physical activity, * p < .05; 415 observations from 296 individuals (nesting of modality of leisure time vs sport). See text for estimated marginal means post-hoc analyses.

**Table 3:** Results of Simple Linear Regression Analysis Testing Whether the Association between Habit Strength and Behaviour Differs Between Team vs Individual Sport

	*b*	95% Confidence Interval
Intercept	161.63*	114.90 to 208.37
Habit strength	0.31*	0.13 to 0.50
Team vs individual sport (with individual as reference)	7.17	-11.23 to 25.57
Habit × team vs individual sport	0.08	-0.22 to 0.40
Age	-2.07*	-3.81 to -0.33
Gender (with male as reference)	14.61	-4.12 to 33.35
Survey language (with English as reference)	-53.89*	-28.85 to -78.92

*Note:* * *p* < .05; *Adj. R^2^* = 0.28, *p* < .01

## Data Availability

Data are available online: 10.17605/OSF.IO/HMA38 of Open Science Framework https://doi.org/10.17605/OSF.IO/HMA38 (Rebar, et al., 2023); Scripts and code are available online: 10.17605/OSF.IO/HMA38 of Open Science Framework https://doi.org/10.17605/OSF.IO/HMA38 (Rebar, et al., 2023); Supplementary information is available online: 10.17605/OSF.IO/HMA38 of Open Science Framework https://doi.org/10.17605/OSF.IO/HMA38 (Rebar, et al., 2023).
